# Fetal growth velocity standards from the Fetal Growth Longitudinal Study of the INTERGROWTH-21^st^ Project

**DOI:** 10.1016/j.ajog.2020.07.054

**Published:** 2021-02

**Authors:** Eric O. Ohuma, José Villar, Yuan Feng, Luo Xiao, Laurent Salomon, Fernando C. Barros, Leila Cheikh Ismail, William Stones, Yasmin Jaffer, Manuela Oberto, J. Alison Noble, Michael G. Gravett, Qingqing Wu, Cesar G. Victora, Ann Lambert, Paola Di Nicola, Manorama Purwar, Zulfiqar A. Bhutta, Stephen H. Kennedy, Aris T. Papageorghiou, International Fetal and Newborn Growth Consortium for the 21st Century (INTERGROWTH-21st Project), M. Katz, M.K. Bhan, C. Garza, S. Zaidi, A. Langer, P.M. Rothwell, Sir D. Weatherall, Z.A. Bhutta, J. Villar, S. Kennedy, D.G. Altman, F.C. Barros, E. Bertino, F. Burton, M. Carvalho, L. Cheikh Ismail, W.C. Chumlea, M.G. Gravett, Y.A. Jaffer, A. Lambert, P. Lumbiganon, J.A. Noble, R.Y. Pang, A.T. Papageorghiou, M. Purwar, J. Rivera, C. Victora, J. Villar, D.G. Altman, Z.A. Bhutta, L. Cheikh Ismail, S. Kennedy, A. Lambert, J.A. Noble, A.T. Papageorghiou, J. Villar, S. Kennedy, L. Cheikh Ismail, A. Lambert, A.T. Papageorghiou, M. Shorten, L. Hoch, H.E. Knight, E.O. Ohuma, C. Cosgrove, I. Blakey, D.G. Altman, E.O. Ohuma, J. Villar, D.G. Altman, F. Roseman, N. Kunnawar, S.H. Gu, J.H. Wang, M.H. Wu, M. Domingues, P. Gilli, L. Juodvirsiene, L. Hoch, N. Musee, H. Al-Jabri, S. Waller, C. Cosgrove, D. Muninzwa, E.O. Ohuma, D. Yellappan, A. Carter, D. Reade, R. Miller, A.T. Papageorghiou, L. Salomon, A. Leston, A. Mitidieri, F. Al-Aamri, W. Paulsene, J. Sande, W.K.S. Al-Zadjali, C. Batiuk, S. Bornemeier, M. Carvalho, M. Dighe, P. Gaglioti, N. Jacinta, S. Jaiswal, J.A. Noble, K. Oas, M. Oberto, E. Olearo, M.G. Owende, J. Shah, S. Sohoni, T. Todros, M. Venkataraman, S. Vinayak, L. Wang, D. Wilson, Q.Q. Wu, S. Zaidi, Y. Zhang, P. Chamberlain, D. Danelon, I. Sarris, J. Dhami, C. Ioannou, C.L. Knight, R. Napolitano, S. Wanyonyi, C. Pace, V. Mkrtychyan, L. Cheikh Ismail, W.C. Chumlea, F. Al-Habsi, Z.A. Bhutta, A. Carter, M. Alija, J.M. Jimenez-Bustos, J. Kizidio, F. Puglia, N. Kunnawar, H. Liu, S. Lloyd, D. Mota, R. Ochieng, C. Rossi, M. Sanchez Luna, Y.J. Shen, H.E. Knight, D.A. Rocco, I.O. Frederick, Z.A. Bhutta, E. Albernaz, M. Batra, B.A. Bhat, E. Bertino, P. Di Nicola, F. Giuliani, I. Rovelli, K. McCormick, R. Ochieng, R.Y. Pang, V. Paul, V. Rajan, A. Wilkinson, A. Varalda, B. Eskenazi, L.A. Corra, H. Dolk, J. Golding, A. Matijasevich, T. de Wet, J.J. Zhang, A. Bradman, D. Finkton, O. Burnham, F. Farhi, F.C. Barros, M. Domingues, S. Fonseca, A. Leston, A. Mitidieri, D. Mota, I.K. Sclowitz, M.F. da Silveira, R.Y. Pang, Y.P. He, Y. Pan, Y.J. Shen, M.H. Wu, Q.Q. Wu, J.H. Wang, Y. Yuan, Y. Zhang, M. Purwar, A. Choudhary, S. Choudhary, S. Deshmukh, D. Dongaonkar, M. Ketkar, V. Khedikar, N. Kunnawar, C. Mahorkar, I. Mulik, K. Saboo, C. Shembekar, A. Singh, V. Taori, K. Tayade, A. Somani, E. Bertino, P. Di Nicola, M. Frigerio, G. Gilli, P. Gilli, M. Giolito, F. Giuliani, M. Oberto, L. Occhi, C. Rossi, I. Rovelli, F. Signorile, T. Todros, W. Stones, M. Carvalho, J. Kizidio, R. Ochieng, J. Shah, S. Vinayak, N. Musee, C. Kisiang’ani, D. Muninzwa, Y.A. Jaffer, J. Al-Abri, J. Al-Abduwani, F.M. Al-Habsi, H. Al-Lawatiya, B. Al-Rashidiya, W.K.S. Al-Zadjali, F.R. Juangco, M. Venkataraman, H. Al-Jabri, D. Yellappan, S. Kennedy, L. Cheikh Ismail, A.T. Papageorghiou, F. Roseman, A. Lambert, E.O. Ohuma, S. Lloyd, R. Napolitano, C. Ioannou, I. Sarris, M.G. Gravett, C. Batiuk, M. Batra, S. Bornemeier, M. Dighe, K. Oas, W. Paulsene, D. Wilson, I.O. Frederick, H.F. Andersen, S.E. Abbott, A.A. Carter, H. Algren, D.A. Rocco, T.K. Sorensen, D. Enquobahrie, S. Waller

**Affiliations:** a Centre for Tropical Medicine and Global Health, Headington, Oxford, United Kingdom; b Maternal, Adolescent, Reproductive & Child Health (MARCH) Centre, London School of Hygiene & Tropical Medicine (LSHTM), London, United Kingdom; c Nuffield Department of Women’s & Reproductive Health, University of Oxford, Oxford, United Kingdom; d Oxford Maternal & Perinatal Health Institute, Green Templeton College, University of Oxford, Oxford, United Kingdom; e Department of Statistics, North Carolina State University, Raleigh, NC; f Department of Obstetrics and Fetal Medicine, Hôpital Necker Enfants Malades, Université Paris Descartes, Paris, France; g Programa de Pós-Graduação em Saúde e Comportamento, Universidade Católica de Pelotas, Pelotas, Brazil; h Clinical Nutrition and Dietetics Department, University of Sharjah, Sharjah, United Arab Emirates; i Faculty of Health Sciences, Aga Khan University, Nairobi, Kenya; j Department of Family & Community Health, Ministry of Health, Muscat, Sultanate of Oman; k S.C. Ostetricia 2U, Città della Salute e della Scienza di Torino, Italy; l Department of Engineering Science, Institute of Biomedical Engineering, University of Oxford, Oxford, United Kingdom; m Departments of Obstetrics & Gynecology and Public Health, University of Washington, Seattle, WA; n Department of Ultrasound, Beijing Obstetrics and Gynecology Hospital, Capital Medical University, Beijing, China; o Programa de Pós-Graduação em Epidemiologia, Universidade Federal de Pelotas, Pelotas, Brazil; p Dipartimento di Scienze Pediatriche e dell’ Adolescenza, Terapia Intensiva Neonatale Ospedale (TINO), Torino, Italy; q Nagpur INTERGROWTH-21st Research Centre, Ketkar Hospital, Nagpur, India; r Center for Global Child Health, Hospital for Sick Children, Toronto, Canada

**Keywords:** correlation models, fetal growth, fetal growth velocity, fetal velocity standards, longitudinal study

## Abstract

**Background:**

Human growth is susceptible to damage from insults, particularly during periods of rapid growth. Identifying those periods and the normative limits that are compatible with adequate growth and development are the first key steps toward preventing impaired growth.

**Objective:**

This study aimed to construct international fetal growth velocity increment and conditional velocity standards from 14 to 40 weeks’ gestation based on the same cohort that contributed to the INTERGROWTH-21^st^ Fetal Growth Standards.

**Study Design:**

This study was a prospective, longitudinal study of 4321 low-risk pregnancies from 8 geographically diverse populations in the INTERGROWTH-21^st^ Project with rigorous standardization of all study procedures, equipment, and measurements that were performed by trained ultrasonographers. Gestational age was accurately determined clinically and confirmed by ultrasound measurement of crown-rump length at <14 weeks’ gestation. Thereafter, the ultrasonographers, who were masked to the values, measured the fetal head circumference, biparietal diameter, occipitofrontal diameter, abdominal circumference, and femur length in triplicate every 5 weeks (within 1 week either side) using identical ultrasound equipment at each site (4–7 scans per pregnancy). Velocity increments across a range of intervals between measures were modeled using fractional polynomial regression.

**Results:**

Peak velocity was observed at a similar gestational age: 16 and 17 weeks’ gestation for head circumference (12.2 mm/wk), and 16 weeks’ gestation for abdominal circumference (11.8 mm/wk) and femur length (3.2 mm/wk). However, velocity growth slowed down rapidly for head circumference, biparietal diameter, occipitofrontal diameter, and femur length, with an almost linear reduction toward term that was more marked for femur length. Conversely, abdominal circumference velocity remained relatively steady throughout pregnancy. The change in velocity with gestational age was more evident for head circumference, biparietal diameter, occipitofrontal diameter, and femur length than for abdominal circumference when the change was expressed as a percentage of fetal size at 40 weeks’ gestation. We have also shown how to obtain accurate conditional fetal velocity based on our previous methodological work.

**Conclusion:**

The fetal skeleton and abdomen have different velocity growth patterns during intrauterine life. Accordingly, we have produced international Fetal Growth Velocity Increment Standards to complement the INTERGROWTH-21^st^ Fetal Growth Standards so as to monitor fetal well-being comprehensively worldwide. Fetal growth velocity curves may be valuable if one wants to study the pathophysiology of fetal growth. We provide an application that can be used easily in clinical practice to evaluate changes in fetal size as conditional velocity for a more refined assessment of fetal growth than is possible at present (https://lxiao5.shinyapps.io/fetal_growth/). The application is freely available with the other INTERGROWTH-21^st^ tools at https://intergrowth21.tghn.org/standards-tools/.

## Introduction

Fetal anthropometric measurements, assessed by ultrasound scanning during pregnancy, are taken as an indirect means of assessing fetal size. Values are plotted on one of the many reference charts available, which have been developed using a variety of methods and varying scientific rigor.^1^^,^^2^ Size measures at the extreme ends (eg, below the 3rd, 5th, or 10th centiles, or above the 90th, 95th, or 97th centiles) of an often locally derived reference distribution are typically interpreted as markers of growth impairment for the purpose of identifying fetuses at an increased risk of adverse perinatal outcomes.

**AJOG at a Glance**

**Why was this study conducted?**

This study aimed to identify fetuses at risk of adverse outcomes because actual rates of skeletal and organ growth differ across time, and insults at different time points during pregnancy almost certainly have differential effects on growth.

**Key findings**

We present fetal velocity standards using fetal data collected prospectively in a rigorous scientific manner from low-risk women, whose newborns have been followed until 2 years of age. We provide an application that can be used easily in clinical practice to evaluate changes in fetal size as conditional velocity for a more refined assessment of fetal growth than is possible at present.

**What does this add to what is known?**

These standards may be valuable if one wants to study the pathophysiology of fetal growth comprehensively. They perfectly complement our existing fetal growth standards (distance), which are already being used clinically in many settings.

However, size and growth are not synonymous terms—a fact that is frequently ignored or misunderstood.^3^, ^4^, ^5^, ^6^ Size is an individual measure taken at a specific point in time; repeated size measures represent distant variations in size. In contrast, growth is a change in a measure per unit of time—hence, a dynamic process.^3^^,^^7^ Specific charts for each objective should be purposely derived from several anthropometric measures obtained longitudinally from the same fetuses and expressed as distance or velocity measures.^5^^,^^8^, ^9^, ^10^ Charts should conform to the recommendations of the World Health Organization (WHO) for monitoring human growth and be based on the prescriptive approach, that is, they should be international standards, derived from healthy populations that have minimal nutritional, environmental, or socioeconomic constraints on growth.^11^

To our knowledge, the only published international fetal growth charts that conform completely to the WHO prescriptive recommendations are those constructed using data from the INTERGROWTH-21^st^ Fetal Growth Longitudinal Study (FGLS).^12^, ^13^, ^14^ However, the use of such distance growth charts in clinical practice may not be sufficient to identify fetuses at a risk of adverse outcomes because (1) actual rates of skeletal and organ growth differ across time, and (2) insults at different time points during pregnancy almost certainly have differential effects on the growth and development of the skeleton and individual organs.^15^ It should, therefore, be self-evident that the concept of the differential growth velocity of fetal structures is in conflict with the practice of using single summary indicators of fetal growth, such as estimated fetal weight (EFW). To illustrate the point, poor placental nutrient transfer in the second trimester of pregnancy leads to early-onset fetal growth restriction (FGR) including impaired skeletal growth,^16^ whereas in later pregnancy, it leads to the depletion of fetal fat stores.^17^

Thus, to complement the existing international INTERGROWTH-21^st^ Fetal Growth (*Distance)* Standards,^12^ we present here international Fetal Growth (*Velocity Increment and Conditional Velocity)* Standards, based on the same serial ultrasound measures obtained from the FGLS cohort. We also provide an easy-to-use application (app) that enables assessment of velocity increment and conditional velocity for fetal head circumference (HC), biparietal diameter (BPD), occipitofrontal diameter (OFD), abdominal circumference (AC), and femur length (FL) (https://lxiao5.shinyapps.io/fetal_growth/). The app is freely available with the other INTERGROWTH-21^st^ tools at https://intergrowth21.tghn.org/standards-tools/.

## Materials and Methods

### Design

INTERGROWTH-21^st^ was a multicenter, population-based project carried out between 2009 and 2016 in 8 delimited urban areas: Pelotas, Brazil; Turin, Italy; Muscat, Oman; Oxford, United Kingdom; Seattle, WA; Shunyi County, a suburban district of the Beijing municipality, China; the central area of the city of Nagpur (Central Nagpur), Maharashtra, India; and the Parklands suburb of Nairobi, Kenya.^12^ At each study site, we recruited women with no clinically relevant obstetrical, gynecologic, or medical history, who initiated antenatal care <14^+0^ weeks’ gestation by menstrual dates and met the entry criteria of optimal health, nutrition, education, and socioeconomic status. This resulted in a group of educated, affluent, clinically healthy women, with adequate nutritional status, who by definition were at a low risk of FGR and preterm birth. A detailed description of the entry criteria and definitions has been published previously.^12^

The last menstrual period (LMP) was used to calculate gestational age provided that (1) the date was certain, (2) the woman had a regular 24- to 32-day menstrual cycle, (3) she had not been using hormonal contraception or breastfeeding in the preceding 2 months, and (4) any discrepancy between the gestational ages based on LMP and crown-rump length (CRL), measured by ultrasound at 9^+0^ to 13^+6^ weeks from LMP was ≤7 days, using the formula described by Robinson and Fleming.^18^ To ensure that CRL measures were interpreted consistently, the Robinson and Fleming formula was loaded into all study ultrasound machines; whenever another machine had to be used locally for CRL measurement, a conversion table extracted from the same formula was provided. The CRL technique was also standardized across sites, and all ultrasonographers were uniformly trained.^19^

FGLS was 1 of the 9 component studies of the INTERGROWTH-21^st^ Project, which has been described in detail elsewhere.^12^, ^13^, ^14^^,^^20^ In brief, FGLS involved performing serial examinations with the same ultrasound machine (Philips HD-9, Philips Ultrasound, USA with curvilinear abdominal transducers C5-2, C6-3, V7-3) every 5 weeks (within 1 week either side) after an initial scan at <14 weeks’ gestation that confirmed the certain clinical dates; hence, the possible ranges of scan visits were at 14 to 18, 19 to 23, 24 to 28, 29 to 33, 34 to 38, and 39 to 42 weeks’ gestation. At each visit after 14 weeks’ gestation, the fetal measures obtained were HC, BPD, OFD, AC, and FL. Each parameter was measured in triplicate from 3 separately obtained images of each structure. These studies have provided robust evidence of the similarities in skeletal growth from early pregnancy to 2 years of age in the infants of healthy women, irrespective of ancestry, and have now been extended beyond skeletal growth to neurodevelopment.^14^^,^^20^

The measurement protocol, including masking the ultrasonographer to the values, and the unique training, standardization, and quality control procedures have been reported elsewhere.^21^, ^22^, ^23^, ^24^ In brief, ultrasonographers were recruited based on their technical experience, motivation, reliability, and ability to speak the local languages. They underwent rigorous training consisting of acquiring theoretical knowledge and familiarity with the study protocol, ultrasound machine and operations manual, and data collection and quality control measures. Centralized hands-on training and initial standardization were also conducted.^12^ In addition, site-specific standardization was conducted at regular intervals by the Ultrasound Quality Control Unit, based in Oxford, to ensure proper use of the ultrasound equipment, calibration, and adherence to the protocol. A quality control system was implemented throughout the study based on (1) assessing the distributions of the 3 masked measurements taken for HC, BPD, OFD, AC, and FL at each scan; and (2) the Ultrasound Quality Control Unit taking a random 10% sample of all ultrasound images, assessing their quality using a validated scoring system, and remeasuring them.^24^ Only after 3 measurements of each structure were recorded was each average value revealed to the ultrasonographer for clinical purposes. The reproducibility of the fetal ultrasound measurements has been previously reported.^23^

The cohort enrolled in FGLS was followed up to 2 years of age and evaluated for their skeletal growth, nutrition, health, and the WHO gross motor milestones.^20^^,^^25^

The INTERGROWTH-21^st^ Project was approved by the Oxfordshire Research Ethics Committee C (reference: 08/H0606/139), the research ethics committees of the individual participating institutions, and the corresponding regional health authorities where the project was implemented. Participants provided written consent to be involved in the study. All documentation, protocols, data collection forms, and clinical tools are freely available on the INTERGROWTH-21st website (https://intergrowth21.tghn.org/).

### Statistical methodology

The decision to pool the data from all the study sites to construct fetal velocity increment standards was based on our detailed, previously published analyses of the same data,^14^ using the strategy recommended in the WHO Multicenter Growth Reference Study,^26^ that produced the WHO Child Growth Standards.^27^ Our overall aim was to produce velocity increments that change smoothly with gestational age and maximize simplicity without compromising model fit; we have, in addition, produced fetal conditional velocity standards. The general strategy and statistical considerations for the analysis of the FGLS data are described in detail elsewhere.^28^^,^^29^

### Velocity increment

Velocity increment was calculated as the difference between 2 ultrasound measures denoted by Y_1_ and Y_2_, divided by the time interval between them, that is, *t*_1_, and *t*_2_, respectively.^30^, ^31^, ^32^, ^33^ The velocity increment rate of growth per week is as follows:

Equation 1 $$\text{Velocity increment}=\left({\text{Y}}_{2}-{\text{Y}}_{1}\right)/\left(t_{2}-t_{1}\right)\;\text{mm}/\text{wk}$$

Velocity increments per week were modeled as a function of gestational age at the mid–time point between any pair of observations on a continuous scale using fractional polynomial regression.^34^ To account for increasing variability with gestational age, the mean and standard deviation (SD) were modeled separately using fractional polynomial regression^34^ of the best fitting powers for HC, BPD, OFD, AC, and FL. To determine velocity increments, we analyzed pairs of observations taken during the course of the serial ultrasound examinations performed every 5 weeks (within 1 week either side).

Goodness of fit incorporated visual inspection of overall model fit by comparing empirical centiles (calculated per complete week of gestation, eg, 38 weeks=38^+0^ to 38^+6^) to the fitted centiles, using quantile-quantile (q-q) plot of the residuals, plots of residuals vs fitted values, and the distribution of fitted Z-scores across gestational ages.

The fitted models were used to obtain velocity centiles on the relative change over each gestational week. Velocity increments were computed as the average relative change for the average week-specific measurement. These velocities were determined across gestational ages from 16 to 40 weeks and for each fetal biometry.

### Conditional velocity

In the context of this paper, we considered conditional velocity as the rate of growth (often referred to as growth velocity) that evaluates velocity based on the change in relative attained size between 2 time points.^33^^,^^35^ A velocity Z-score of 0 denotes perfect tracking, whereas a score above or below 0 represents faster or slower growth than expected between the specified times. However, an important consideration is the well-known phenomenon of regression to the mean,^36^ as many, but not all, small fetuses will on average catch up and many, but not all, large fetuses will “catch down.”^37^ Regression to the mean has far-reaching implications^36^^,^^38^^,^^39^ not often accounted for, especially when assessing velocity. The correlation coefficient is a direct measure of regression to the mean.^37^^,^^38^^,^^40^ The conditional SD scores (cSDSs) account for regression to the mean by adjusting for the correlation between the 2 time points.^37^ The statistical methodology that separately modeled the same data and produced estimates of the correlation between any pair of fetal HC, BPD, OFD, AC, or FL measures between 14 and 40 weeks’ gestation has recently been published.^41^ In brief, to account for nonnormality of fetal measurements, we applied a 2-stage approach. The first stage involved finding a suitable transformation of the raw fetal measurements, as the marginal distributions of ultrasound measurements were nonnormal using Cole’s Lambda (λ), Mu (µ) and Sigma (σ) (LMS) transformation^42^ of 3 parameters (location, scale, and skewness using Box-Cox Cole-Green distribution^42^) and 4 parameters (location, scale, skewness, and kurtosis using Box-Cox *t-*distribution^43^ and Box-Cox power exponential distribution^44^) to standardized deviations (Z-scores). In the second stage, a correlation model for a Gaussian process was fitted, yielding a correlation for any pair of observations made between 14 and 40 weeks of gestation. To model correlations, parametric and nonparametric models were used. Four exponential parametric models were applied, and because growth measurements might have nonignorable measurement errors, a nugget effect term for the exponential model was also explored along with 2 nonparametric models for modeling correlation. Further details are presented in a previous report.^41^ We used the correlation coefficients from this work to calculate the fetal conditional velocity for HC, BPD, OFD, AC, and FL using the cSDS approach.^37^

The FGLS data were converted to Z-scores using the published international INTERGROWTH-21^st^ Fetal Growth (*Distance)* Standards derived from the same data.^12^ Let fetal biometry Z-scores be denoted by Z_1_ and Z_2_ at time points *t*_1_ and *t*_2_, respectively, and *r*_*12*_ the correlation coefficient between Z_1_ and Z_2_. The cSDS between the 2 time points is given by:^38^

Equation 2 $$\text{cSDS}=\left({\text{Z}}_{2}-r_{12}\times {\text{Z}}_{1}\right)/\sqrt{\left(1-r_{12}^{2}\right)},$$

where *t*_1_<*t*_2_.^37^

All analyses were performed in STATA software, version 11.2 (StataCorp LP, College Station, TX) and R statistical software (R Foundation for Statistical Computing).

## Results

### Overall results

In the original FGLS, a total of 4321 women had live singleton births in the absence of severe maternal conditions or congenital abnormalities detected by ultrasound or at birth; this forms the included study sample. The median number of ultrasound scans (excluding the dating scan) was 5.0 (mean, 4.9; SD, 0.8; range, 4–7), and 97% of women had ≥4 scans (mean, 5.0; SD, 0.6; range, 4–7), indicating that the participants adhered well to the protocol. The same population was used for this analysis.

The high protocol adherence meant that the intervals between adjacent measurements were mostly 4 weeks (n=3836), 5 weeks (n=8871), or 6 weeks (n=2411), or intervals involving a combination or multiples of the 4-, 5-, and 6-week intervals: 8 weeks (n=721), 9 weeks (n=2817), 10 weeks (n=5186), 11 weeks (n=1932), and 12 weeks (n=356). In total, 20,030 fetal measures were used to construct the Fetal Growth Velocity Standards.

**Figure 1:**
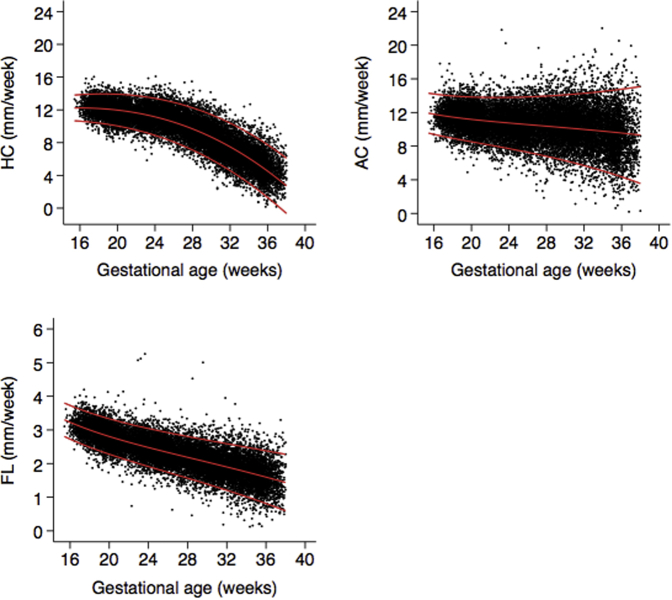
Increments in fetal HC, AC, and FL (mm/wk) according to gestational age (weeks) for all of the sites combined The fitted 3rd, 50th, and 97th centiles are superimposed. *AC*, abdominal circumference; *FL*, femur length; *HC*, head circumference. *Ohuma et al. Fetal growth velocity standards. Am J Obstet Gynecol 2021*.

**Supplemental Figure 1:**
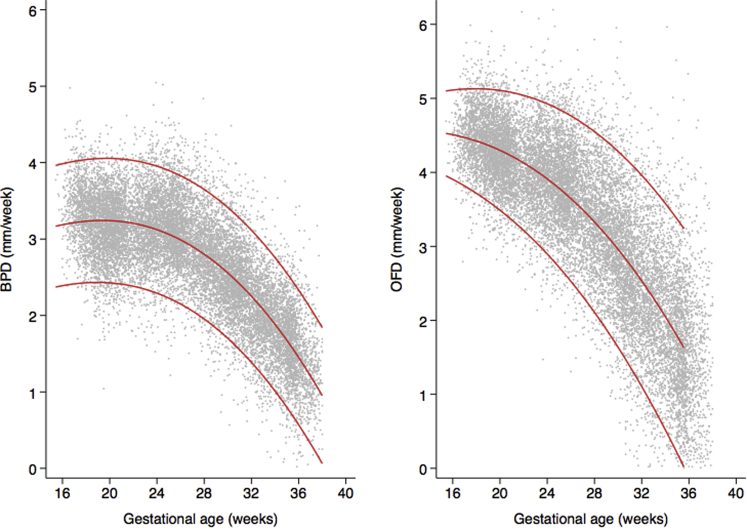
Increments in BPD and OFD (mm/wk) according to gestational age (weeks) for all of the sites combined The fitted 3rd, 50th, and 97th centiles are superimposed. Adapted from Papageorghiou et al.^12^ *BPD*, biparietal diameter; *OFD*, occipitofrontal diameter. *Ohuma et al. Fetal growth velocity standards. Am J Obstet Gynecol 2021*.

A scatterplot of increments in raw HC, AC, BPD, OFD, and FL data (mm/wk) and the fitted 3rd, 50th, and 97th smoothed centiles according to gestational age (weeks) is shown in Figure 1 and Supplemental Figure 1.

The baseline characteristics of the study cohort across the 8 urban areas previously have been shown to be very similar, which was expected because women were selected using the same clinical and demographic criteria (Supplemental Table). The pregnancy and perinatal events for the complete cohort, which confirmed their status as healthy women at low risk of impaired fetal growth, have also been published before.^12^ In addition, the infant cohort remained healthy with adequate growth, motor development, and associated behaviors up to 2 years of age,^20^^,^^45^ supporting its appropriateness for the construction of the INTERGROWTH-21st Fetal Growth (*Distance)* Standards^12^ and associated Preterm Postnatal Growth Standards.^46^

### Velocity increments

The rate of growth for HC was the highest at 16 and 17 weeks’ gestation (12.2 mm/wk), and the velocity slowed down with an almost linear reduction (9.7 mm/wk at 28 weeks vs 6.1 mm/wk at 34 weeks’ gestation) toward term (Figure 1; Table 1). For BPD, peak velocity was observed at 19 and 20 weeks’ gestation (3.2 mm/wk) (Supplemental Figure 1, Table 2). OFD had an earlier observed peak velocity at 16 weeks’ gestation (4.5 mm/wk) (Supplemental Figure 1; Table 3). A similar pattern of growth was seen with the other skeletal measure (FL). The rate of FL growth was the highest very early in pregnancy at 16 weeks’ gestation (mean, 3.2 mm/wk), which reduced to 2.2 mm/wk at 28 weeks and 1.8 mm/wk at 34 weeks’ gestation (Figure 1; Table 4). FL velocity decreased linearly with increasing gestational age.

**Table 1:** Smoothed centiles for FHC velocity increment (mm/wk) according to gestational age

Gestational age (wk)	FHC						
	C3	C5	C10	C50	C90	C95	C97
16	10.62	10.82	11.14	12.24	13.34	13.65	13.85
17	10.52	10.73	11.06	12.22	13.37	13.70	13.91
18	10.39	10.62	10.96	12.17	13.38	13.72	13.94
19	10.23	10.47	10.83	12.09	13.35	13.71	13.94
20	10.04	10.28	10.66	11.97	13.29	13.67	13.91
21	9.81	10.06	10.45	11.82	13.20	13.59	13.84
22	9.54	9.80	10.21	11.64	13.06	13.47	13.73
23	9.24	9.51	9.93	11.41	12.89	13.32	13.59
24	8.89	9.18	9.61	11.15	12.68	13.12	13.40
25	8.51	8.80	9.26	10.85	12.43	12.89	13.18
26	8.08	8.39	8.86	10.50	12.14	12.61	12.91
27	7.62	7.93	8.41	10.11	11.81	12.29	12.61
28	7.10	7.43	7.93	9.68	11.43	11.93	12.25
29	6.55	6.88	7.39	9.20	11.01	11.52	11.86
30	5.94	6.29	6.82	8.68	10.54	11.07	11.41
31	5.29	5.65	6.19	8.11	10.02	10.57	10.92
32	4.60	4.96	5.52	7.49	9.46	10.02	10.39
33	3.85	4.22	4.80	6.82	8.85	9.43	9.80
34	3.05	3.44	4.03	6.11	8.19	8.78	9.16
35	2.21	2.60	3.21	5.34	7.48	8.08	8.48
36	1.31	1.71	2.34	4.52	6.71	7.34	7.74
37	0.36	0.77	1.41	3.65	5.90	6.54	6.95
38	-0.64	-0.22	0.44	2.73	5.03	5.68	6.11

*C*, centile; *FHC*, fetal head circumference.

*Ohuma et al. Fetal growth velocity standards. Am J Obstet Gynecol 2021*.

**Table 2:** Smoothed centiles for fetal BPD velocity increment (mm/wk) according to gestational age

Gestational age (wk)	BPD						
	C3	C5	C10	C50	C90	C95	C97
16	2.4	2.5	2.6	3.2	3.7	3.9	4.0
17	2.4	2.5	2.7	3.2	3.8	3.9	4.0
18	2.4	2.5	2.7	3.2	3.8	3.9	4.0
19	2.4	2.5	2.7	3.2	3.8	4.0	4.1
20	2.4	2.5	2.7	3.2	3.8	4.0	4.1
21	2.4	2.5	2.7	3.2	3.8	3.9	4.1
22	2.4	2.5	2.7	3.2	3.8	3.9	4.0
23	2.3	2.5	2.6	3.2	3.7	3.9	4.0
24	2.3	2.4	2.6	3.1	3.7	3.9	4.0
25	2.2	2.3	2.5	3.1	3.6	3.8	3.9
26	2.2	2.3	2.4	3.0	3.6	3.7	3.8
27	2.1	2.2	2.3	2.9	3.5	3.6	3.8
28	2.0	2.1	2.2	2.8	3.4	3.5	3.7
29	1.8	1.9	2.1	2.7	3.3	3.4	3.5
30	1.7	1.8	2.0	2.6	3.1	3.3	3.4
31	1.6	1.7	1.8	2.4	3.0	3.2	3.3
32	1.4	1.5	1.7	2.3	2.8	3.0	3.1
33	1.2	1.3	1.5	2.1	2.7	2.8	3.0
34	1.0	1.1	1.3	1.9	2.5	2.7	2.8
35	0.8	0.9	1.1	1.7	2.3	2.4	2.6
36	0.6	0.7	0.9	1.5	2.1	2.2	2.3
37	0.3	0.4	0.6	1.2	1.8	2.0	2.1
38	0.1	0.2	0.4	1.0	1.6	1.7	1.8

*BPD*, biparietal diameter; *C*, centile.

*Ohuma et al. Fetal growth velocity standards. Am J Obstet Gynecol 2021*.

**Table 3:** Smoothed centiles for fetal OFD velocity increment (mm/wk) according to gestational age

Gestational age (wk)	OFD						
	C3	C5	C10	C50	C90	C95	C97
16	3.9	4.0	4.1	4.5	4.9	5.0	5.1
17	3.8	3.9	4.0	4.5	4.9	5.0	5.1
18	3.7	3.8	3.9	4.4	4.9	5.0	5.1
19	3.6	3.7	3.9	4.4	4.9	5.0	5.1
20	3.5	3.6	3.8	4.3	4.9	5.0	5.1
21	3.4	3.5	3.6	4.2	4.8	5.0	5.1
22	3.2	3.3	3.5	4.1	4.8	4.9	5.0
23	3.1	3.2	3.4	4.0	4.7	4.9	5.0
24	2.9	3.0	3.2	3.9	4.6	4.8	4.9
25	2.7	2.9	3.1	3.8	4.5	4.7	4.9
26	2.5	2.7	2.9	3.7	4.4	4.6	4.8
27	2.3	2.5	2.7	3.5	4.3	4.5	4.7
28	2.1	2.3	2.5	3.3	4.2	4.4	4.6
29	1.9	2.0	2.3	3.2	4.0	4.3	4.4
30	1.6	1.8	2.1	3.0	3.9	4.1	4.3
31	1.4	1.6	1.8	2.8	3.7	4.0	4.1
32	1.1	1.3	1.6	2.5	3.5	3.8	4.0
33	0.8	1.0	1.3	2.3	3.3	3.6	3.8
34	0.5	0.7	1.0	2.1	3.1	3.4	3.6
35	0.2	0.4	0.7	1.8	2.9	3.2	3.4
36	−0.1	0.1	0.4	1.5	2.6	2.9	3.1
37	−0.5	−0.3	0.1	1.2	2.4	2.7	2.9
38	−0.8	−0.6	−0.3	0.9	2.1	2.4	2.6

*C*, centile; *OFD*, occipitofrontal diameter.

*Ohuma et al. Fetal growth velocity standards. Am J Obstet Gynecol 2021*.

**Table 4:** Smoothed centiles for fetal FL velocity increment (mm/wk) according to gestational age

Gestational age (wk)	FL						
	C3	C5	C10	C50	C90	C95	C97
16	2.7	2.8	2.9	3.2	3.6	3.7	3.7
17	2.6	2.7	2.8	3.1	3.5	3.6	3.6
18	2.5	2.6	2.7	3.0	3.4	3.5	3.5
19	2.4	2.5	2.6	2.9	3.3	3.4	3.4
20	2.3	2.4	2.5	2.8	3.2	3.3	3.3
21	2.2	2.3	2.4	2.7	3.1	3.2	3.3
22	2.1	2.2	2.3	2.6	3.0	3.1	3.2
23	2.0	2.1	2.2	2.6	2.9	3.0	3.1
24	1.9	2.0	2.1	2.5	2.9	3.0	3.0
25	1.8	1.9	2.0	2.4	2.8	2.9	3.0
26	1.7	1.8	1.9	2.3	2.7	2.9	2.9
27	1.7	1.7	1.9	2.3	2.7	2.8	2.9
28	1.6	1.6	1.8	2.2	2.6	2.7	2.8
29	1.5	1.6	1.7	2.1	2.6	2.7	2.8
30	1.4	1.5	1.6	2.0	2.5	2.6	2.7
31	1.3	1.4	1.5	2.0	2.4	2.6	2.6
32	1.2	1.3	1.4	1.9	2.4	2.5	2.6
33	1.1	1.2	1.3	1.8	2.3	2.5	2.5
34	1.0	1.1	1.3	1.8	2.3	2.4	2.5
35	0.9	1.0	1.2	1.7	2.2	2.3	2.4
36	0.8	0.9	1.1	1.6	2.1	2.3	2.4
37	0.7	0.8	1.0	1.5	2.1	2.2	2.3
38	0.6	0.7	0.9	1.4	2.0	2.2	2.3

*C*, centile; *FL*, femur length.

*Ohuma et al. Fetal growth velocity standards. Am J Obstet Gynecol 2021*.

Conversely, the velocity growth for AC (consisting of abdominal organs and subcutaneous fat) was relatively steady across most gestational ages, from 16 weeks (mean, 11.8 mm/wk) to 10.4 mm/wk at 28 weeks and 9.7 mm/wk at 34 weeks’ gestation. This pattern is clearly different from that of HC (Figure 1; Table 5).

**Table 5:** Smoothed centiles for AC velocity increment (mm/wk) according to gestational age

Gestational age (wk)	AC						
	C3	C5	C10	C50	C90	C95	C97
16	9.4	9.7	10.1	11.8	13.4	13.9	14.2
17	9.1	9.5	9.9	11.6	13.3	13.8	14.1
18	8.9	9.2	9.7	11.5	13.2	13.7	14.0
19	8.7	9.1	9.6	11.3	13.1	13.6	13.9
20	8.5	8.9	9.4	11.2	13.0	13.5	13.9
21	8.3	8.7	9.2	11.1	12.9	13.5	13.8
22	8.1	8.5	9.0	11.0	12.9	13.5	13.8
23	7.9	8.3	8.9	10.9	12.9	13.4	13.8
24	7.7	8.1	8.7	10.8	12.8	13.4	13.8
25	7.5	7.9	8.5	10.7	12.8	13.4	13.8
26	7.3	7.7	8.3	10.6	12.8	13.5	13.9
27	7.0	7.5	8.1	10.5	12.8	13.5	13.9
28	6.8	7.2	7.9	10.4	12.8	13.5	14.0
29	6.5	7.0	7.7	10.3	12.8	13.6	14.0
30	6.3	6.8	7.5	10.2	12.8	13.6	14.1
31	6.0	6.5	7.3	10.1	12.9	13.7	14.2
32	5.7	6.2	7.0	10.0	12.9	13.7	14.3
33	5.4	5.9	6.8	9.9	12.9	13.8	14.4
34	5.0	5.6	6.5	9.7	13.0	13.9	14.5
35	4.7	5.3	6.3	9.6	13.0	14.0	14.6
36	4.3	5.0	6.0	9.5	13.0	14.1	14.7
37	3.9	4.6	5.7	9.4	13.1	14.2	14.8
38	3.5	4.2	5.3	9.2	13.2	14.3	15.0

*AC*, abdominal circumference; *C*, centile.

*Ohuma et al. Fetal growth velocity standards. Am J Obstet Gynecol 2021*.

**Figure 2:**
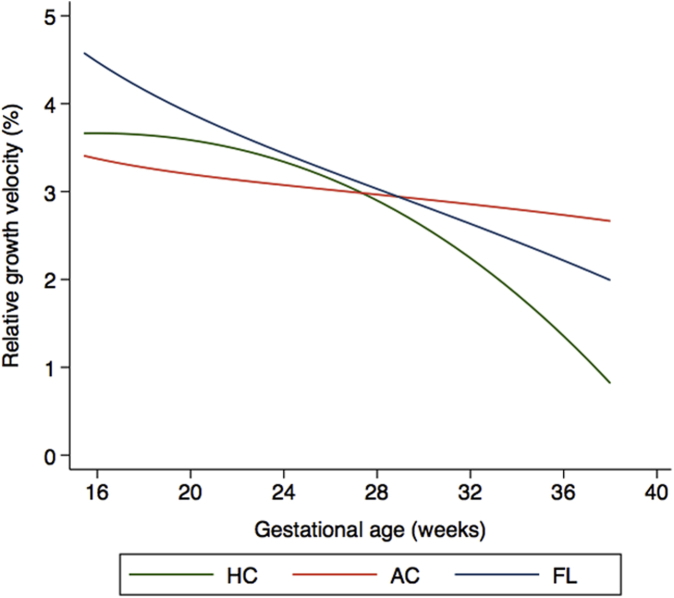
Median fetal velocity increments for HC (green), AC (red), and FL (blue) expressed as a percentage of attained fetal size at 40 weeks’ according to INTERGROWTH-21^st^ Fetal Standards^12^ Adapted from Papageorghiou et al.^12^ *AC*, abdominal circumference; *FL*, femur length; *HC*, head circumference; *INTERGROWTH-21st*, International Fetal and Newborn Growth Consortium for the 21st Century. *Ohuma el al. Fetal growth velocity standards. Am J Obstet Gynecol 2021*.

Figure 2 shows the velocity increment growth presentations of fetal HC, AC, and FL relative to the expected attained size at 40 weeks’ gestation according to the published international INTERGROWTH-21^st^ Fetal Growth (*Distance)* Standards.^12^ It is clear that 90% (30.2 cm at 33 weeks and 33.4 cm at 40 weeks) of the HC size at term was reached by 33 weeks’ gestation (Figure 2).

**Supplemental Figure 2:**
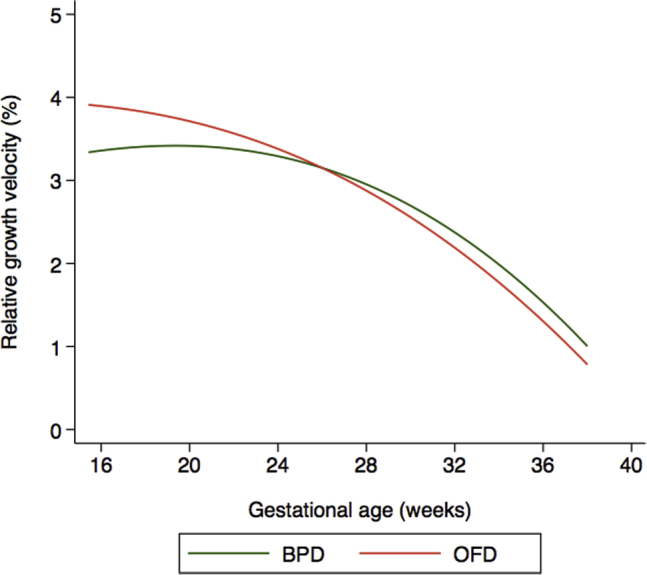
Median velocity increments in BPD (green) and OFD (red) according to gestational age expressed as a percentage of the attained fetal size at 40 weeks’ gestation according to the published international INTERGROWTH-21st Fetal (Distance) Growth Standards *BPD*, biparietal diameter; *INTERGROWTH-21st*, International Fetal and Newborn Growth Consortium for the 21st Century; *OFD*, occipitofrontal diameter. Adapted from Papageorghiou et al.^12^ *Ohuma et al. Fetal growth velocity standards. Am J Obstet Gynecol 2021*.

The change in velocity with gestational age was more clearly seen in the skeletal markers for HC, BPD, OFD, and FL than AC when expressed as a percentage of size at 40 weeks’ gestation^12^ (Figure 2; Supplemental Figure 2). AC gain was steady at around 3% per week (range, 2.7%–3.4%) of the total size at term; HC gain was close to 4% of the term size per week at 16 weeks and <1% after 36 weeks’ gestation (range, 0.8%–3.7%). FL gain was highest in early pregnancy and decreased linearly with advancing gestational age (range, 2%–4.5%).

Tables 1, 2, 3, 4, and 5 present the predicted 3rd, 5th, 10th, 50th, 90th, 95th, and 97th centiles for velocity increments between 14 and 40 weeks’ gestation for HC, BPD, OFD, AC, and FL, respectively, to match the previously published Fetal Growth *(Distance)* Standards.^12^ The corresponding equations for the mean and SD from the fractional polynomial regression models for each measure are presented in Table 6, allowing for calculations by readers of any desired centiles or Z-scores according to gestational age. For example, centiles can be calculated as mean±Z×SD, where Z is −1.88, −1.645, −1.28, 0, 1.28, 1.645, and 1.88 for the 3rd, 5th, 10th, 50th, 90th, 95th, and 97th centiles, respectively. Printable charts and related tools will be available free of any charge at http://www.intergrowth.org.uk.

**Table 6:** Equations for the estimation of the expected mean and SD velocity increment (mm/wk) for each fetal biometry measurement according to gestational age

Fetal biometry	Measurement	Regression equation
HC	Mean	10.43136+1.377907×((GA/10)^2^)+(−1.431528×((GA/10)^2^×log(GA/10)))
	SD	0.1791373+(0.0425136×(GA))
BPD	Mean	2.263092+0.6066072×((GA/10)^2^)+(−0.5224027×((GA/10)^2^×log(GA/10)))
	SD	0.3886744+(0.0022155×(GA))
OFD	Mean	4.308462+0.2489315×((GA/10)^2^)+(−0.3629665×((GA/10)^2^×log(GA/10)))
	SD	−0.1167106+(0.0273527×(GA))
AC	Mean	10.56711+3.392895×((GA/10)^−2^)+(−0.0285397×((GA/10)^3^))
	SD	1.137471+(0.0349324×(GA/10)^3^)
FL	Mean	1.474157+2.899183×((GA/10)^−1^)+(−0.0147426×((GA/10)^3^))
	SD	0.2507282+(0.0035916×(GA/10)^3^)

All log are natural logarithms.

Centiles can be calculated as mean±z×SD, where z=−1.88, −1.645, −1.28, 0, 1.28, 1.645, and 1.88 for the 3rd, 5th, 10th, 50th, 90th, 95th, and 97th centiles, respectively, which are represented as C3, C5 C10, C50, C90, C95, and C97 in Tables 1, 2, 3, 4, and 5.

*AC*, abdominal circumference, *BPD*, biparietal diameter; *FL*, femur length; *GA*, gestational age; *HC*, head circumference; *OFD*, occipitofrontal diameter; *SD*, standard deviation.

*Ohuma et al. Fetal growth velocity standards. Am J Obstet Gynecol 2021*.

### Conditional velocity

**Figure 3:**
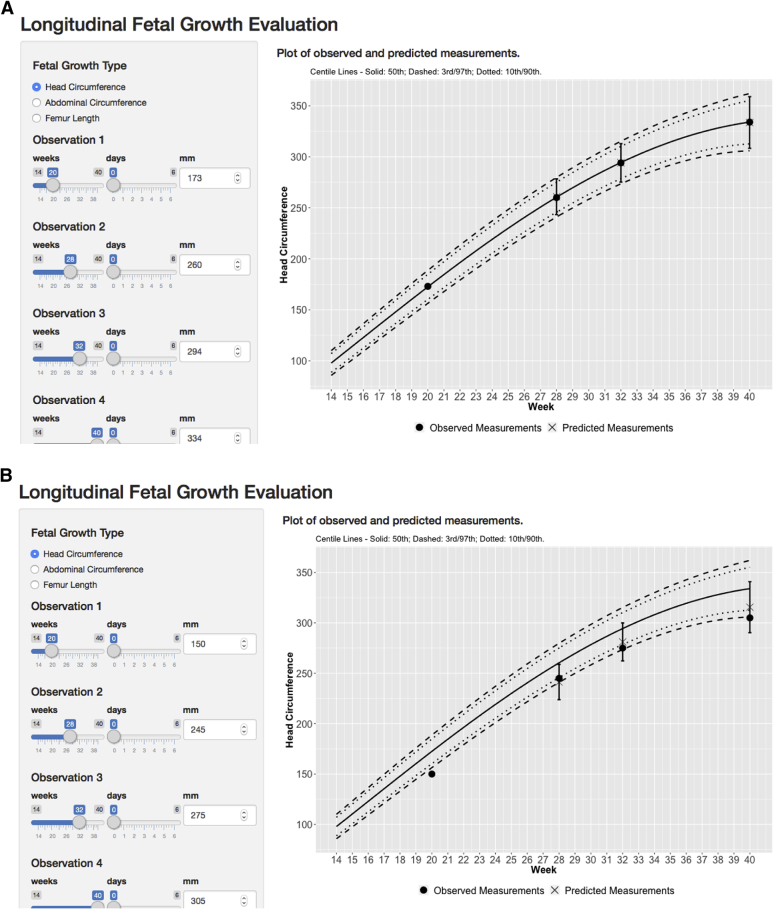

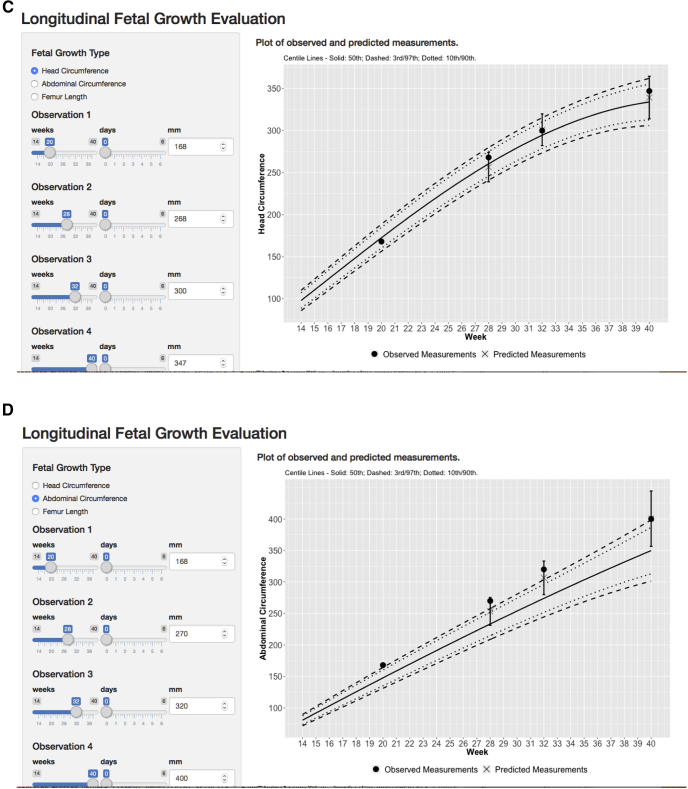
Screenshots of the fetal velocity app showing 4 example plots of longitudinal fetal growth evaluation based on observed and predicted measurements for a fetus **A**, Normal fetus based on head circumference. **B,** Possible microcephaly based on head circumference. **C,** A fetus within 2 standard deviations. **D,** Possible case of macrosomia based on abdominal circumference. All measurements were compared with the published international INTERGROWTH-21st Fetal (Distance) Growth Standards. *INTERGROWTH-21st*, International Fetal and Newborn Growth Consortium for the 21st Century. Adapted from Papageorghiou et al.^12^ *Ohuma et al. Fetal growth velocity standards. Am J Obstet Gynecol 2021*.

We randomly selected measures across different gestational ages and used the fitted correlations and observed Z-scores^12^ to illustrate conditional velocity (cSDS) for a single fetus according to gestational age. For demonstration purposes, we show in Figure 3, A–D, 4 hypothetical fetal HC growth scenarios likely to be observed during pregnancy: a fetus that exhibits the expected average rate of growth throughout pregnancy (scenario A), a fetus whose longitudinal pattern of growth exhibits possible microcephaly (scenario B), a fetus whose pattern of growth is within 2 SD of an established fetal HC standard (scenario C), and a fetus whose longitudinal pattern of growth exhibits possible macrosomia (scenario D).

These calculations and visual illustrations are embedded in the R-shiny app (https://lxiao5.shinyapps.io/shinycalculator/). In addition, the app converts fetal measures to Z-scores according to the international Fetal Growth *(Distance)* Standards of the INTERGROWTH-21^st^ Project^12^; enables correlations to be calculated between any pair of fetal biometry measures to calculate conditional velocity (cSDS); and calculates velocity increments for HC, BPD, OFD, AC, and FL (https://lxiao5.shinyapps.io/shinycalculator/).

## Comment

### Principal findings

We described growth velocity increment and estimated velocity standards and conditional velocity using highly standardized ultrasound measures of the skeleton and abdominal organs or fat of fetuses from the FGLS cohort of the INTERGROWTH-21^st^ Project. This is the same cohort from which not only the INTERGROWTH-21^st^ Fetal Growth *(Distance)* Standards^12^ but also the international standards for the following were developed: (1) symphysis-fundal height,^47^ (2) gestational weight gain,^48^ (3) early and late pregnancy dating,^49^ (4) EFW,^50^ (5) newborn body composition,^51^ and (6) postnatal growth of preterm infants.^46^ Hence, the new velocity standards allow, for the first time, distance and velocity growth in utero to be assessed using longitudinal growth trajectories from the same international populations of pregnant women at a low risk for adverse health and nutritional and environmental factors, that is, prescriptive populations.

In addition, for the first time in the obstetrical ultrasound literature, we followed up the cohort until 2 years of age and showed that the mean skeletal growth of the infants participating in FGLS was well within the WHO Child Growth Standards (50th centile for HC, 49th centile for length, and 58th centile for weight). These findings strongly suggest that the fetal growth velocity increments described here are likely to be observed in healthy populations worldwide.

### Results

Our results show that peak growth velocity was observed between 16 and 17 weeks’ gestation for the fetal skeleton (HC and FL) and abdomen (AC); however, the observed patterns were markedly different. OFD had an earlier peak velocity at 16 weeks than 19 and 20 weeks’ gestation for BPD, which implies that the fetal head may have a rate of growth that promotes a slightly disproportionate shape to accommodate certain brain structures.

Growth velocity slowed down rapidly for HC and FL and at an almost linear rate in the case of FL; by contrast, it remained steady throughout pregnancy for AC. In addition, there was a larger variability in the AC velocity values than those for HC and FL, especially in the second half of pregnancy. Taken together, the findings show that overall skeletal growth is a biological process that has the highest rate of growth in the first part of pregnancy; linear skeletal growth (FL) is even more pronounced.

How do our results compare with previously published studies? For HC, Deter et al,^52^ using the Rossavik growth model in a cohort of 20 fetuses, reported an earlier peak velocity at 14 weeks (14 mm/wk), which decreased to 9 mm/wk at 30 weeks and 5 mm/wk at 38 weeks’ gestation.^53^ Similarly, for AC, peak velocity was earlier (12 mm/wk at 14 weeks and reduced to 11 mm/wk at 30 weeks of gestation). Todros et al,^54^ applying a growth model similar to Rossavik’s found that the peak velocity was at around 16 weeks’ gestation for both HC and BPD. Similarly, Guihard-Costa et al^55^ reported multiphasic patterns of growth velocity, with a common peak velocity at about 16 weeks of gestation and no sex differences in growth velocity. Bertino et al^56^ reported similar findings to ours: in 238 fetuses, peak velocity was reached at 17.3 weeks’ gestation with a rapid increase in the early part of the second trimester, which then decreased up until the end of pregnancy.

Several studies using different selection criteria, hospital populations, ultrasound equipment, and methodologies have reported a decrease in FL linear growth velocity by gestational age.^57^, ^58^, ^59^ However, it is important to bear in mind that the populations studied were not equivalent and that FL is measured differently by modern equipment; hence, the values are not entirely comparable.^60^ This is an important issue because hospitals are still using FL charts based on equipment that is no longer in use, which increases the risk of misclassifying fetuses.

Recently, Grantz et al^61^ studied the relationship between fetal growth velocity and self-reported maternal ethnicity. The findings were similar to those of this study: FL velocity was between 3.4 and 3.5 mm/wk at 16 weeks (3.2 mm/wk in our study), 2.2 mm/wk at 28 weeks (2.2 mm/wk in our study), and between 1.8 and 1.9 mm/wk at 34 weeks’ gestation (1.8 mm/wk in our study). Therefore, as the INTERGROWTH-21^st^ Project has clearly demonstrated, fetal linear skeletal growth velocity seems to be very similar regardless of the mother’s geographic location, country of origin, or self-reported ethnicity, which adds considerable support to the concept that growth among healthy, low-risk fetuses is universal.^62^

AC, which is an indicator of abdominal organ growth (mostly liver and subcutaneous fat), is strongly influenced by the underlying nutritional status of the population being studied. In developed countries and in countries suffering from the double burden of malnutrition, where a large proportion of the population is in the midst of the obesity epidemic, it is becoming increasingly clear that overweight or obesity is often initiated in utero.^63^ Hence, comparing AC growth in previous studies^54^^,^^55^ with the FGLS cohort of healthy, low-risk women is a less relevant question given that fetal AC values in unselected populations are influenced by the distribution of maternal fat–related markers, that is, the fetal AC may be larger in obese women than in those with a normal body mass index.^64^

Grantz et al,^61^ in a US population, reported AC velocity values early in pregnancy that were very similar to our study: 11.7 to 12.2 mm/wk at 16 weeks (11.8 mm/wk in our study) and 10.3 to 10.9 mm/wk at 28 weeks’ gestation (10.4 mm/wk in our study). However, in the third trimester, there was clear evidence of AC overgrowth: 10.1 to 10.8 mm/wk at 34 weeks’ gestation vs 9.7 mm/wk in our study. In short, fat may be deposited in the fetal abdomen faster in some populations than in others, resulting in overweight, despite similar skeletal growth velocities.

### Clinical implications

Our analysis of skeletal and abdominal velocity increments, expressed as a percentage of attained fetal size at 40 weeks’ gestation, also showed differential growth velocity patterns. This finding has important clinical consequences because of how EFW is utilized in obstetrical practice. Based on these findings and taking into account basic analytical principles, summary measures such as EFW should not be used if there is evidence of biological heterogeneity among the parameters to be combined. Thus, the observation that similar EFW estimations can be obtained from different permutations of HC, AC, and FL values may explain the large errors often seen in EFW values regardless of which equation is used.^65^^,^^66^

The biological significance of the heterogeneity in the velocity and timing of fetal growth is best appreciated by examining how an intrauterine insult, such as infection with the Zika virus (ZIKV), can have varying effects at different stages of pregnancy. Infection in the first trimester has clearly been associated with massive disruption to brain development and a decrease in the rate of head growth, resulting in microcephaly.^67^ However, brain damage can also arise from infection late in the pregnancy despite head size remaining within the normal limits.^68^^,^^69^ Certainly, in our data set, 90% of HC at term was reached by 33 weeks’ gestation, that is, HC values at birth and at 33 weeks’ gestation were very similar. Therefore, a ZIKV infection after 33 weeks’ gestation may still lead to brain damage but the effect on the skull size will be limited, which means that diagnosing ZIKV infection based solely on the presence of microcephaly at birth will be associated with a false-negative rate. We can extend this type of comparison into childhood. The mean HC of the same cohort at 2 years of age was 47.8 cm; although we acknowledge differences in measurement techniques, on average, two-thirds of a 2-year-old child’s HC is attained by 33 weeks’ gestation.

### Strengths and limitations

This study’s findings could have important implications for clinical practice, as improved assessment of fetal growth patterns could potentially lead to more personalized antenatal care. In other words, the use of the standards described here could help distinguish healthy from disturbed fetal growth for both the management of individual pregnancies and for screening purposes. However, there are practical challenges. A similar approach has been advocated in the past for monitoring child growth; however, it has not been adopted in routine practice largely because the calculations are complex and the results are difficult to interpret. To illustrate the point, the choice of interval length between measures affects the results: the shorter the interval, the higher the variability in growth and measurement error than the actual growth. Guihard-Costa et al^55^ recommend a 3-week interval as the minimum time interval in which the growth rate may be statistically significant, taking into account the number of cases, the minute fluctuations of growth rate in short periods, and the individual variability of growth velocity. However, extending the time interval loses the benefit of assessing velocity especially during the third trimester, when the peak of growth has passed for skeletal markers, AC variability is very large, and birth is soon likely to occur. Frequent ultrasound measurements are also not presently recommended for routine antenatal care and have implications for cost, staff numbers, and workload. Our robust statistical modeling work of the correlation of fetal biometry measurements using a 2-stage approach addressed at least some of these limitations by enabling the calculation of fetal biometry correlations for any pair of observations between 14 and 40 weeks and is independent of time interval.^41^

To facilitate the use of the standards described here, we have provided an easy-to-use R-shiny app (freely available at https://lxiao5.shinyapps.io/fetal_growth/) for assessing conditional velocity if repeat ultrasound measures are clinically indicated. We believe that both distance and velocity assessments of fetal growth would help clinicians to detect fetuses at risk of a growth abnormality. There are clinical advantages of assessing growth using conditional velocity. For instance, a fetus may not meet its growth trajectory, yet not fall below a cutoff centile (such as the 10th); however, a size chart would not identify that fetus as small for gestational age, despite its evident poor growth over time.

### Research implications

This study’s findings offer new avenues for both clinical and life sciences research. It may now be possible to identify more refined fetal growth phenotypes (or “fetotypes”), matching those described for the neonate, which may be associated with certain child health outcomes. Hence, we encourage health professionals worldwide to join us in determining the clinical significance of deviations from optimal skeletal and fat-dependent growth by conducting research to establish if routine fetal growth velocity assessment can improve health outcomes.^70^ External assessment of the findings in daily practice, including the implications of growth above or below the standards, are areas for future research. The potential pathophysiologic significance of the growth velocity patterns identified here should also prompt a renewed focus on research into the underlying cellular and molecular mechanisms responsible for fetal growth.

### Conclusions

We found that fetal growth velocity increment is the highest in the early part of the second trimester and decreases with gestational age for skeletal structures and that there is a clear continuous pattern throughout pregnancy for fat-related markers such as AC. Furthermore, we constructed international Fetal Growth *Velocity Increment* Standards and conditional velocity to complement the set of tools produced by the INTERGROWTH-21^st^ Project.^12^ A free, simple-to-use, online clinical tool is presented here, enabling the calculation of fetal growth velocity throughout pregnancy.

## Figures and Tables

**Supplemental Table:** Baseline characteristics of the FGLS

Characteristic	Pregnancies (N=4321)
Maternal age (y), mean (SD)	28.4 (3.9)
Maternal height (cm), mean (SD)	162.2 (5.8)
Maternal weight (kg), mean (SD)	61.3 (9.1)
Paternal height (cm), mean (SD)	174.4 (7.3)
Body mass index (kg/m^2^), mean (SD)	23.3 (3.0)
Gestational age at first visit (wk), mean (SD)	11.8 (1.4)
Years of formal education (y), mean (SD)	15.0 (2.8)
Hemoglobin level <15^+0^ wk (g/dL), mean (SD)	12.5 (1.1)
Married or cohabiting, n (%)	4204 (97.3)
Nulliparous, n (%)	2955 (68.4)
Preeclampsia, n (%)	31 (0.7)
Pyelonephritis, n (%)	16 (0.4)
Any sexually transmitted infection, n (%)	3 (0.1)
Spontaneous initiation of labor, n (%)	2868 (66.4)
PPROM (<37^+0^ wk), n (%)	80 (1.9)
Cesarean delivery, n (%)	1541 (35.7)
NICU admission >1 d, n (%)	240 (5.6)
Preterm (<37^+0^ wk), n (%)	195 (4.5)
Preterm and spontaneous onset of labor, n (%)	126 (2.9)
Term LBW (<2500 g; ≥37^+0^ wk), n (%)	128 (3.0)
Neonatal mortality, n (%)	7 (0.2)
Male sex, n (%)	2149 (49.7)
Exclusive breastfeeding at discharge, n (%)	3786 (87.6)
Mother admitted to intensive care unit, n (%)	17 (0.4)
Birthweight (kg) (≥37^+0^ wk), mean (SD)	3.3 (0.4)
Birth length (cm) (≥37^+0^ wk), mean (SD)	49.4 (1.9)
Birth head circumference (cm) (≥37^+0^ wk), mean (SD)	33.9 (1.3)

*FGLS*, Fetal Growth Longitudinal Study; *LBW*, low birthweight; *NICU*, neonatal intensive care unit; *PPROM*, preterm prelabor rupture of membranes; *SD*, standard deviation.

*Ohuma et al. Fetal growth velocity standards. Am J Obstet Gynecol 2021*.
